# Supplementary material for: Pharmacological Ascorbate Elicits Anti-Cancer Activities against Non-Small Cell Lung Cancer through Hydrogen-Peroxide-Induced-DNA-Damage
Source: Antioxidants (Basel). 2023 Sep 18;12(9):1775. doi: 10.3390/antiox12091775 (PMC10525775; doi:10.3390/antiox12091775)
Supplement: Supplementary file 1 [file antioxidants-12-01775-s001.zip › antioxidants-2555062-supplementary.pdf]

antioxidants

MDPI

Pharmacological Ascorbate Elicits Anti-Cancer Activities Against Non-Small Cell Lung Cancer Through Hydrogen-Peroxide-Induced-DNA-Damage

Kittipong Sanookpan [1,2], Naphat Chantaravisoot [3,4,5], Nuttiya Kalpongnukul [5,6], Chatchapon Chuenjit [3], Onsurang Wattanathamsan [1], Sara Shoaib [1], Pithi Chanvorachote [1,7] and Visarut Buranasudja [1,8,*]

1. Department of Pharmacology and Physiology, Faculty of Pharmaceutical Sciences, Chulalongkorn University, Bangkok, 10330, Thailand
2. Nabsolute Co., Ltd., Bangkok, 10330, Thailand
3. Department of Biochemistry, Faculty of Medicine, Chulalongkorn University, Bangkok, 10330, Thailand.
4. Center of Excellence in Systems Microbiology, Faculty of Medicine, Chulalongkorn University, Bangkok, 10330, Thailand.
5. Center of Excellence in Systems Biology, Faculty of Medicine, Chulalongkorn University, Bangkok, Thailand, 10330.
6. Research Affairs, Faculty of Medicine, Chulalongkorn University, Bangkok, 10330, Thailand.
7. Cell-Based Drug and Health Product Development Research Unit, Faculty of Pharmaceutical Sciences, Chulalongkorn University, Bangkok, 10330, Thailand
8. Center of Excellence in Natural Products for Ageing and Chronic Diseases, Faculty of Pharmaceutical Sciences, Chulalongkorn University, Bangkok, 10330, Thailand

* Corresponding author: Visarut Buranasudja, M.Sc. Ph.D.; Department of Pharmacology and Physiology, Faculty of Pharmaceutical Sciences, Chulalongkorn University, 254 Phayathai Road, Patumwan, Bangkok 10330 Thailand; Tel.: +66-65-056-5638; E-mail: visarut.b@pharm.chula.ac.th.

Supplementary Information

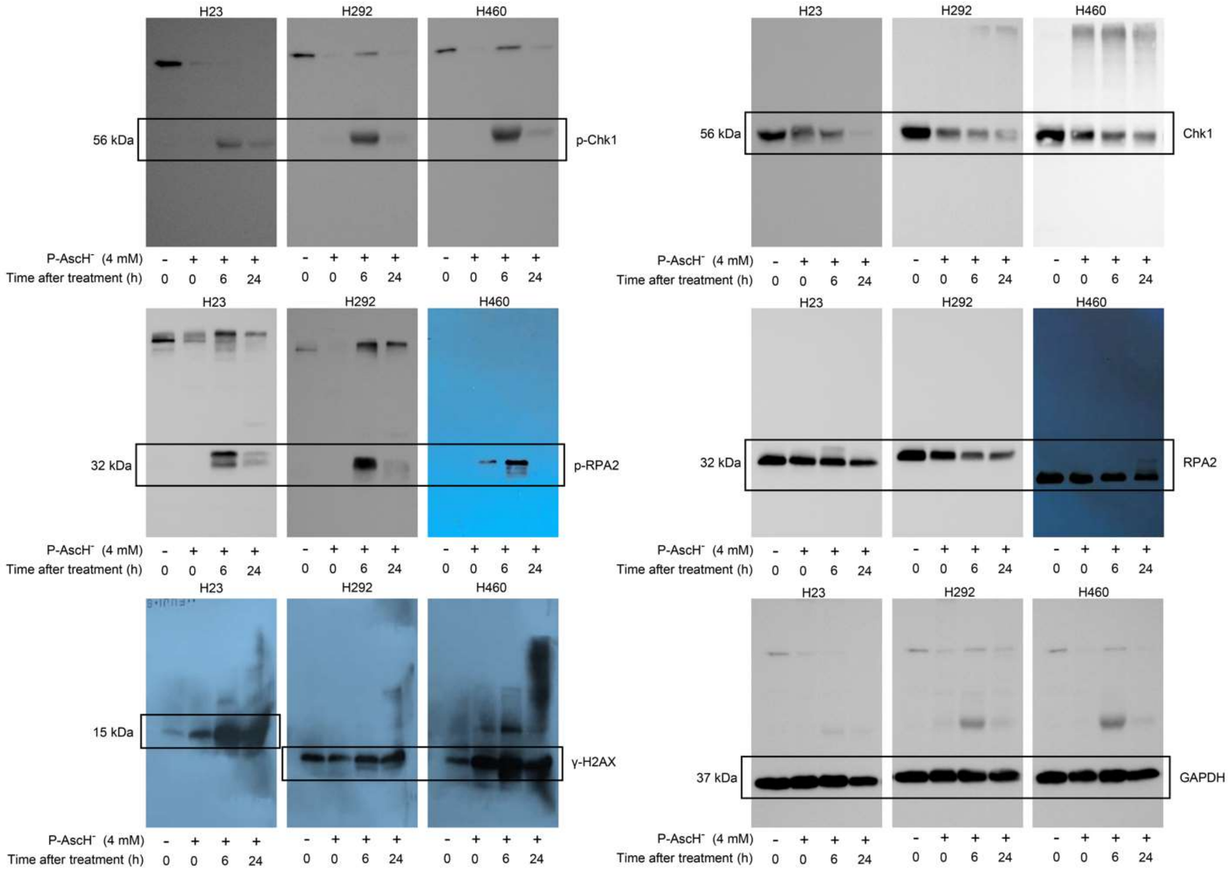

Figure S1. A. Original western blot images. Full unedited western blots for p-Chk1 (56 kDa), Chk1 (56 kDa), p-RPA2 (32 kDa), RPA2 (32 kDa), γ-H2AX (15 kDa) and GAPDH (37 kDa). These western blot images represent three biological replicates and are used to prepare Figure 4.

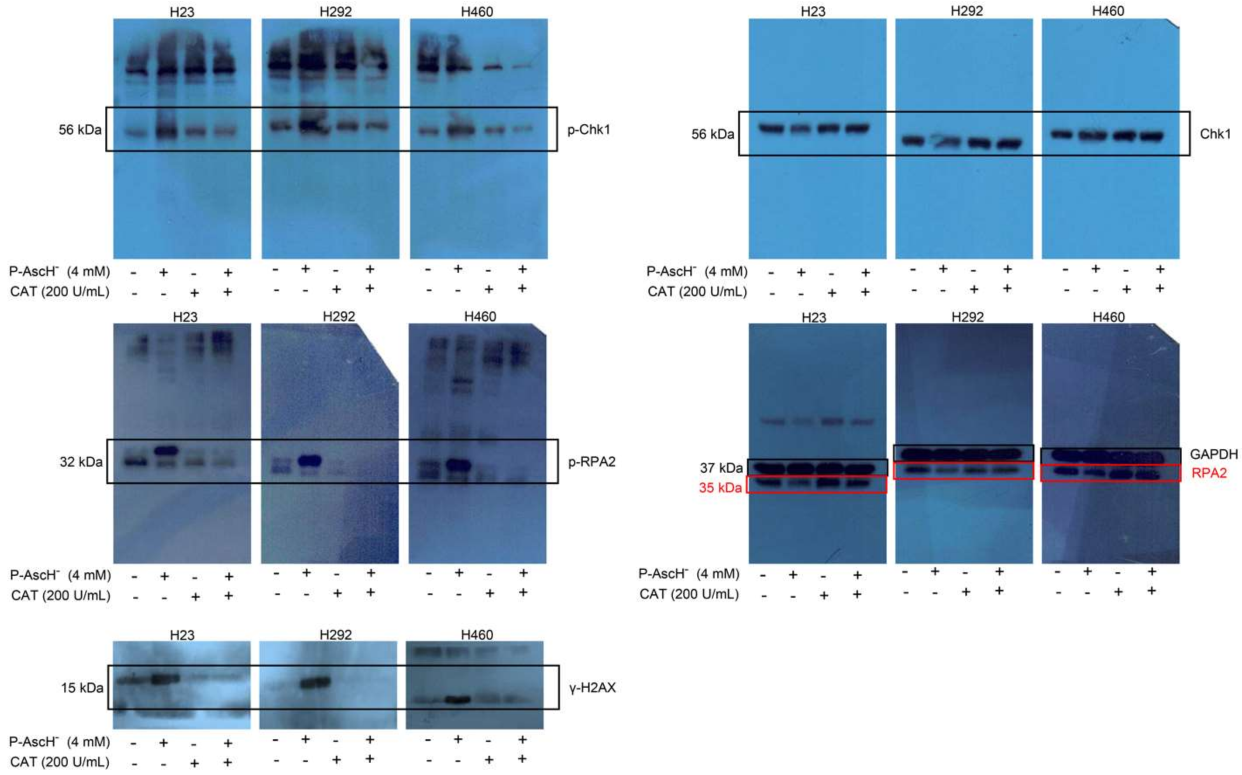

Figure S1. B. Full unedited western blots for p-Chk1 (56 kDa), Chk1 (56 kDa), p-RPA2 (32 kDa), RPA2 (32 kDa), γ-H2AX (15 kDa) and GAPDH (37 kDa). These western blot images represent three biological replicates and are used to prepare Figure 5.

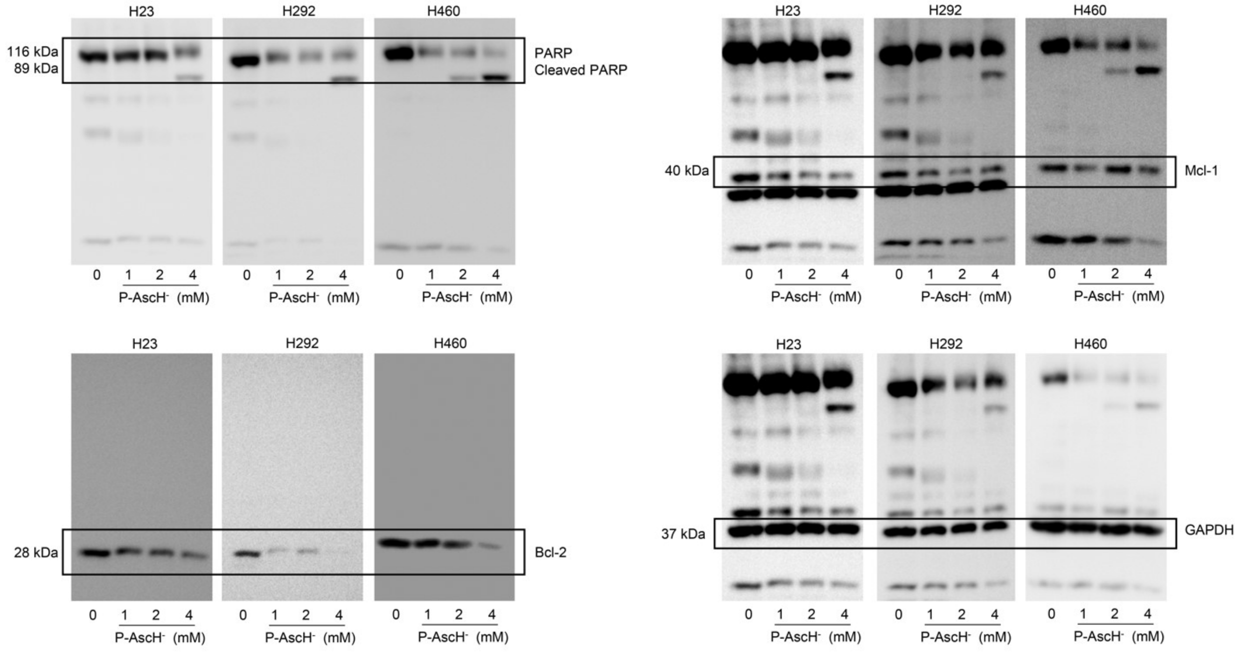

Figure S1. C. Full unedited western blots for PARP (116 kDa), cleaved PARP (89 kDa), Mcl-1 (40 kDa), Bcl-2 (28 kDa), and GAPDH (37 kDa). These western blot images represent three biological replicates and are used to prepare Figure 8.

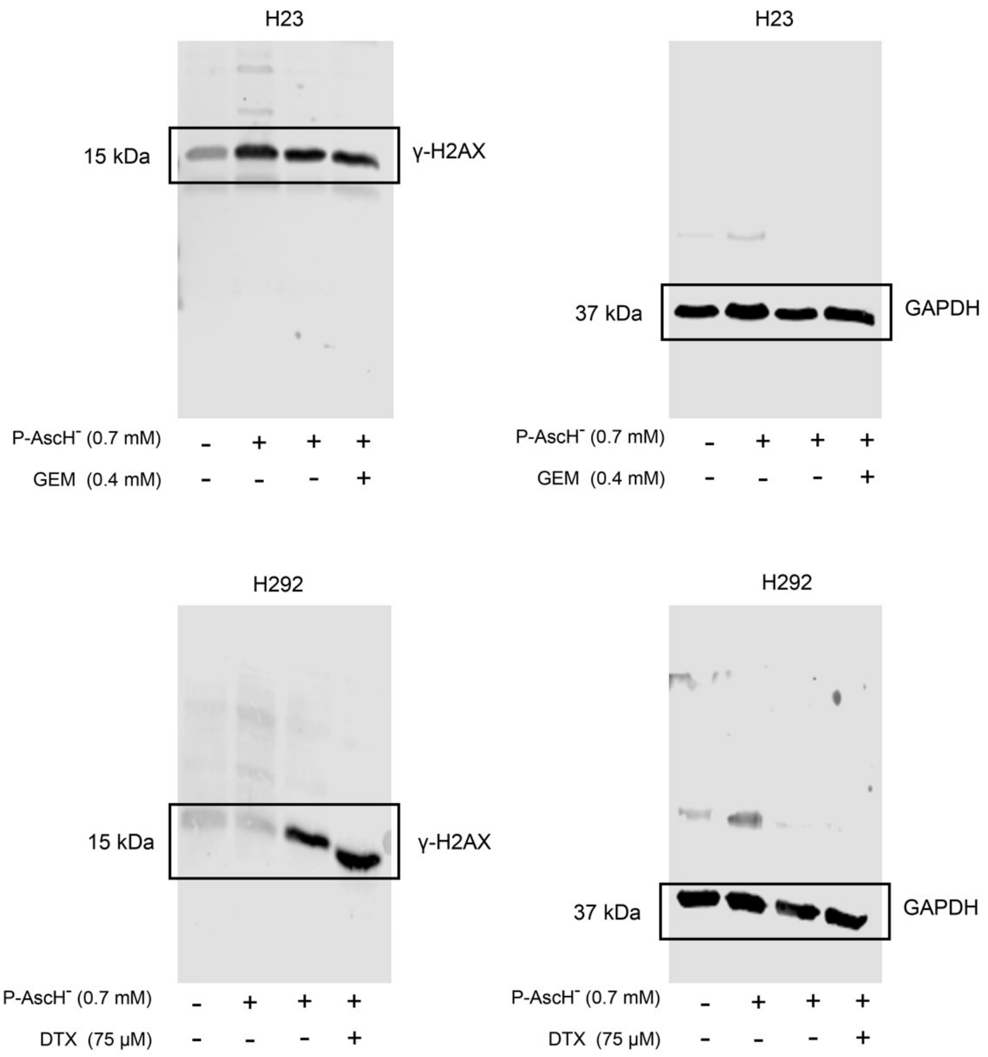

Figure S1. D. Full unedited western blots for γ-H2AX (15 kDa) and GAPDH (37 kDa). These western blot images represent three biological replicates and are used to prepare Figure S5.

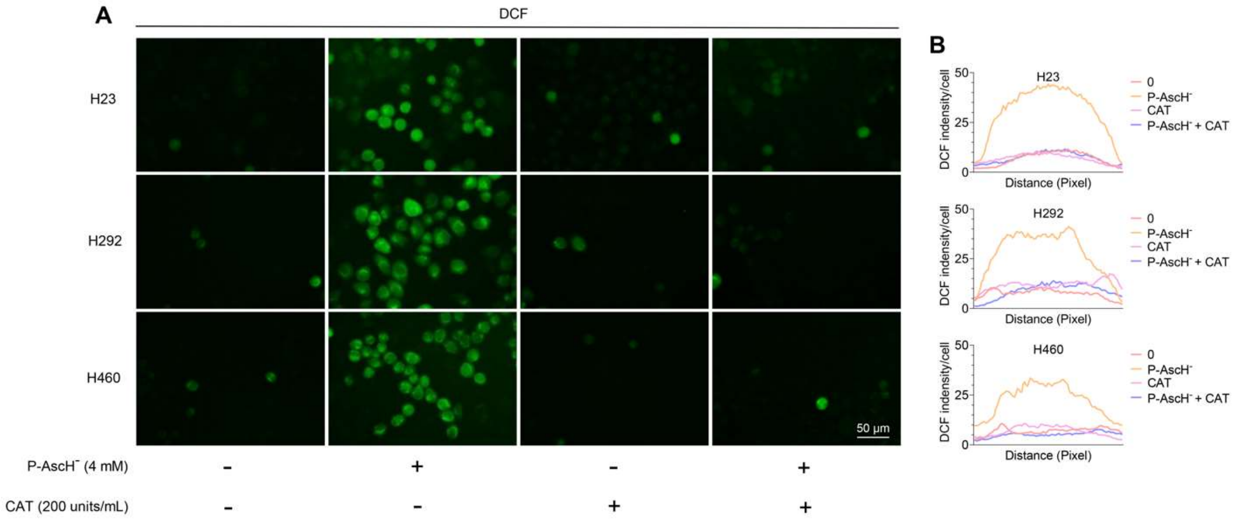

Figure S2. Pharmacological ascorbate induces oxidative distress on NSCLC *via* the formation of extracellular H_2O_2.**A,** Treatments with pharmacological ascorbate resulted in an increased oxidation of DCFH-DA on NSCLC, indicating an induction of oxidative distress. Co-treatment with

extracellular catalase suppressed an induction of oxidative distress upon P-AscH⁻ exposure. Cells were incubated with P-AscH⁻ (4 mM) ± extracellular catalase (200 U/mL) for 1 h. Following treatments, the oxidation of DCFH-DA (green) was observed under fluorescence microscopy. **B**, The graphs demonstrate the fluorescence intensity of A. The densitometric analysis was conducted by using ImageJ software (*n* = 3; magnification, 40x; scale bar, 50 µm). .

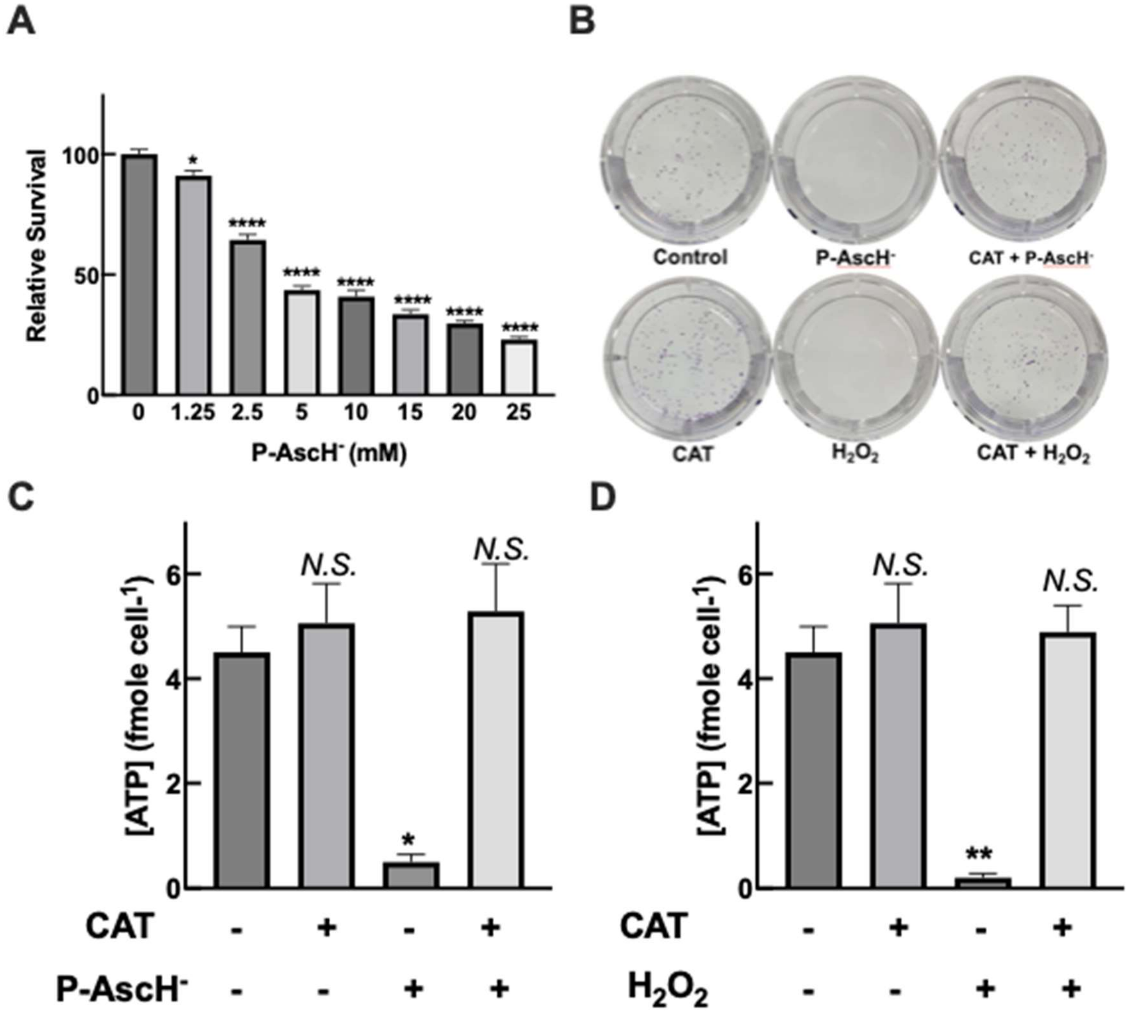

Figure S3. The anti-cancer effects of P-AscH⁻ against A549 cells is mainly due to formation of extracellular H_2O_2. **A,** Treatment with P-AscH⁻ caused reduction in cell survival of A549 in dose-dependent fashion. A549 human lung adenocarcinoma cells were incubated with P-AscH⁻ (0 – 25 mM; 1 h) and the cell viability was observed with MTT at 24-h post-treatment. **B**, Co-treatment with extracellular catalase significantly inhibited P-AscH⁻-induced-clonogenic-cell-death in A549 cells. A549 cells were incubated with P-AscH⁻ (15 mM) ± catalase (200 U/mL) for 1 h and the clonogenic survival assay was conducted to evaluate clonogenicity of cells following treatment. **C and D,** The depletion in ATP storage upon P-AscH⁻ treatment is mainly due to formation of extracellular H_2O_2. A549 cells were exposed to P-AscH⁻ (15 mM) or H_2O_2 (2 mM) ± catalase (200 U/mL) for 1 h, then the intracellular ATP was immediately determined after treatment (*n* = 3; mean ± SEM; *, $p < 0.05$, ***, $p < 0.001$, ****, $p < 0.0001$ *v.s.* untreated control; *N.S.*, no significant) .

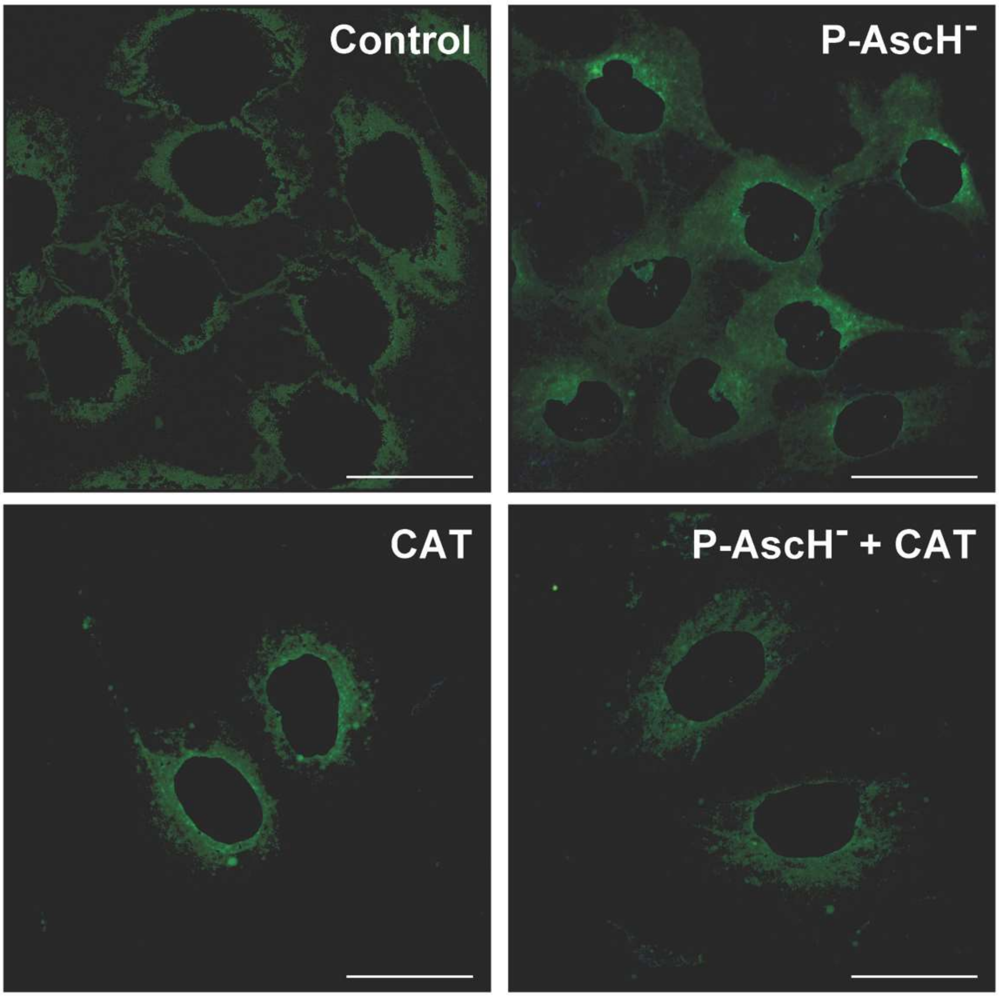

Figure S4. Pharmacological ascorbate enhances cytoplasmic γ-H2AX levels of H460 cells. H460 cells were treated with 4mM P-AsCH$^-$, bovine catalase (200 U/mL), or co-treated with P-AscH$^-$ (4 mM) and catalase (200 U/mL) for a duration of 1 h. Subsequently, immunofluorescence staining was conducted at 6 h post-treatment using anti-γ-H2AX (green) antibodies. Cell nuclei were stained with DAPI. To facilitate the visualization and quantitation of cytoplasmic γ-H2AX, the nuclear regions of individual cells (DAPI positive) were masked. Data is representative of three independent studies. The quantified levels of cytoplasmic γ-H2AX are illustrated in Fig 6B. Scale bar, 20 μm.

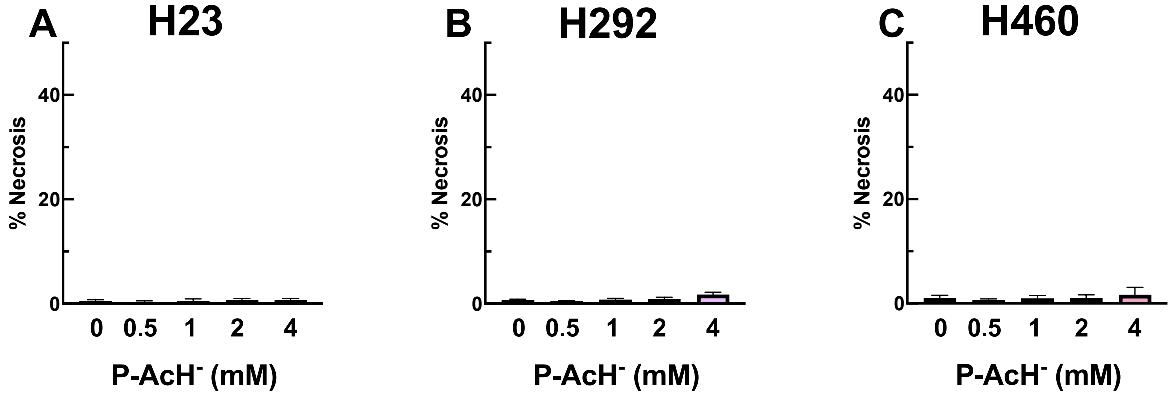

Figure S5. Pharmacological ascorbate does not induce necrotic cell death in NSCLC. A-C, There were no increase in PI-positive cells following P-AscH$^-$ treatments. H292 and H460 cells were treated with P-AscH$^-$ (0 – 4 mM; 1 h) and mode of cell death were characterized with flow cytometric analysis using Annexin V/PI co-staining at 24-h-post treatment.

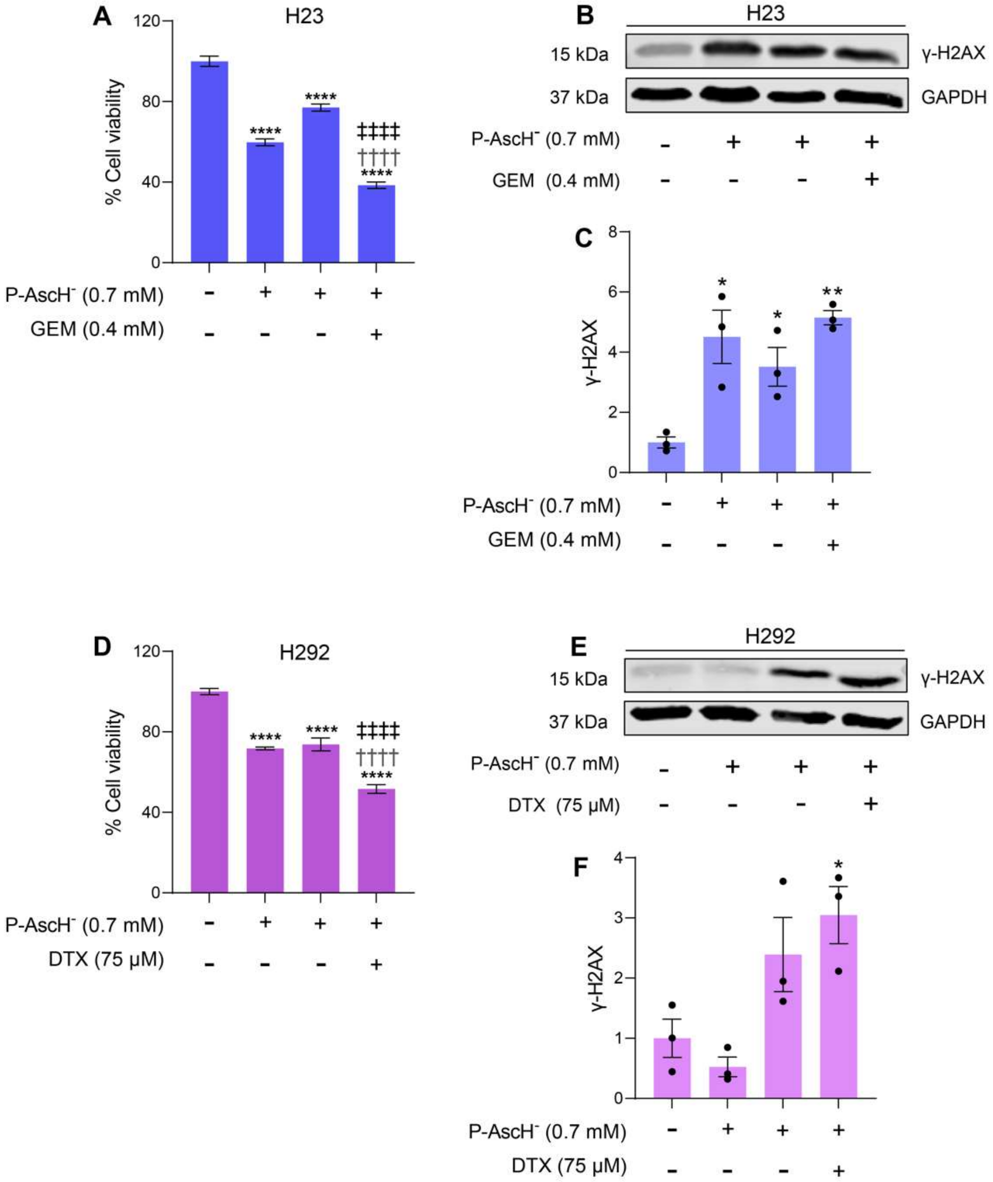

Figure S6. The potential synergistic effects of P-AscH$^-$ on anti-cancer activities of gemcitabine and docetaxel may be attributed to an augmentation of genotoxic effects. H23 cells were treated with gemcitabine (GEM; 0.4 mM; 24 h), or P-AscH$^-$ (0.7 mM; 1 h), or a combination of GEM (0.4 mM; 24 h) + P-AscH$^-$ (0.7 mM; 1 h), while H292 cells were exposed to docetaxel (DTX; 75 μM; 24 h), or P-AscH$^-$ (0.7 mM 1 h), or a combination of DTX (75 μM; 24 h) + P-AscH$^-$ (0.7 mM 1 h). At the 24 h following these treatments, **A and D**, cell viability was assessed with MTT assay (n = 3; mean ± SEM; ****, $p < 0.0001$ v.s. untreated control; ††††, $p < 0.0001$ v.s. P-AscH-; ‡‡‡‡, $p < 0.0001$ v.s. GEM or DTX); **B and E**, the presence of γ-H2aX was determined by western blot analysis; **C and F**, the density of each protein band was quantified and subsequently normalized with GAPDH. The data was presented as fold change relative to the untreated control (n = 3; mean ± SEM; *, $p < 0.05$, **, $p < 0.01$ v.s. untreated control).

Extended Discussions

P-AscH$^-$ has exhibited a synergistic amplification of the anti-cancer potential of chemotherapeutic agents, including gemcitabine (GEM), docetaxel (DTX), as exemplified in Fig 9. Additionally, our investigation has effectively highlighted the susceptibility of DNA to the effects of P-AscH$^-$ (Fig 4-6). We postulated that this vulnerability of DNA potentially underpins the mechanism responsible for the observed synergistic activities. To explore this hypothesis, H23 and H292 cells were subjected to a combined treatment (P-AscH$^-$/GEM or P-AscH$^-$/DTX) that resulted in moderate toxicity (approximately 40 – 50% cytotoxicity; Supplementary Fig S5A, and S5D) and the formation of γ-H2aX with western blot analysis were subsequently observed following treatments.

In H23 cells, both separate administration with P-AscH$^-$ (0.7 mM) and GEM (0.4 mM) induced the generation of γ-H2aX. Furthermore, the P-AscH$^-$/GEM treatment amplified this formation of γ-H2aX, when compared to untreated control (Supplementary Fig S5B, and S5C). Conversely, in H292 cells, the presence of detectable γ-H2aX signal was absent upon P-AscH$^-$single treatment under this experimental condition (Supplementary Fig 5E, and 5F). This absence could potentially be attributed to the insufficiency of P-AscH$^-$ dose (0.7 mM) to significantly stimulate generation of γ-H2aX. Although the administration of P-AscH$^-$ alone did not trigger the generation of γ-H2aX, our western blot results suggests that P-AscH$^-$/DTX combination regimen tends to yield higher levels of γ-H2aX compared to DTX treatment alone (Supplementary Fig S5E, and S5F). Collectively, our preliminary results from H23 and H292 cells suggested that P-AscH$^-$ possesses the capacity to amplify genotoxic impacts of chemotherapeutic drugs, thereby augmenting their overall anti-cancer activities. Nonetheless, it is important to acknowledge that detection of γ-H2aX may not serve as an entirely comprehensive method for quantifying the magnitude of genotoxicity following treatments. The supplementary methods that directly quantify the damage on DNA such as quantitative-PCR-based measurement [1], are essential to enhance the validation of this hypothesis.

Table S1. Parameters for confocal microscopy.

Parameters	Channel 1	Channel 2	Channel 3	Channel 4
Target of Detection	BRCA1	RAP80	γ-H2AX	DNA
Light source	HXP 120 V	HXP 120 V	HXP 120 V	HXP 120 V
Light source intensity	1.00%	1.00%	1.00%	1.00%
Laser wavelength	640 nm: 6.00%	561 nm: 7.00%	488 nm: 5.00%	405 nm: 3.00%
Dye	AF647	AF568	AF488	DAPI
Excitation wavelength	653	577	493	353
Emission wavelength	668	603	517	465
Detection wavelength	645-700	560-640	450-575	400-496

**Imaging Device**	LSM800 Airyscan	LSM800 Airyscan	LSM800 Airyscan	LSM800 Airyscan
**Detector**	Airyscan	Airyscan	Airyscan	Airyscan
**Detector gain**	850 V	850 V	850 V	850 V

Table S2. EGFR and KRAS status of tested NSCLC cell lines.

Cell lines	RRID	EGFR status	KRAS status	References
H292	CVCL_0455	WT	WT	[2, 3]
H460	CVCL_0459	WT	p.Q61H	[4]
H23	CVCL_1547	WT	p.G12C	[4]
A549	CVCL_0023	WT	p.G12S	[4]

Table S3. EC_{50} values for gemcitabine and docetaxel treatments.

Cell lines	EC_{50} to GEM (mM)	EC_{50} to DTX ± SEM (μM)
H292	> 10	171.2 ± 4.9
H460	> 10	78.5 ± 4.0
H23	> 10	105.4 ± 3.1

References

1. Furda, A., Santos, J.H., Meyer, J.N., and Van Houten, B., Quantitative PCR-based measurement of nuclear and mitochondrial DNA damage and repair in mammalian cells. *Methods Mol Biol*, **1105**, 419-437 (2014).
2. da Silva-Oliveira, R.J., Gomes, I.N.F., da Silva, L.S., Lengert, A.V.H., Laus, A.C., Melendez, M.E., Munari, C.C., Cury, F.P., Longato, G.B., and Reis, R.M., Efficacy of Combined Use of Everolimus and Second-Generation Pan-EGRF Inhibitors in KRAS Mutant Non-Small Cell Lung Cancer Cell Lines. *Int J Mol Sci*, **23**(14) (2022).
3. Ranayhossaini, D.J., Lu, J., Mabus, J., Gervais, A., Lingham, R.B., and Fursov, N., EGF potentiation of VEGF production is cell density dependent in H292 EGFR wild type NSCLC cell line. *Int J Mol Sci*, **15**(10), 17686-17704 (2014).
4. Blanco, R., Iwakawa, R., Tang, M., Kohno, T., Angulo, B., Pio, R., Montuenga, L.M., Minna, J.D., Yokota, J., and Sanchez-Cespedes, M., A gene-alteration profile of human lung cancer cell lines. *Hum Mutat*, **30**(8), 1199-1206 (2009).
